# Supplementary material for: Role of a TonB-dependent receptor and an oxygenase in iron-dependent copper resistance in Caulobacter crescentus
Source: J Bacteriol. 2025 Mar 14;207(4):e00493-24. doi: 10.1128/jb.00493-24 (PMC12004943; doi:10.1128/jb.00493-24)
Supplement: Supplemental figures and table — Fig. S1 to S6 and Table S1. [file jb.00493-24-s0001.pdf]

Journal of Bacteriology https://doi.org/10.1128/jb.00493-24

Role of a TonB-dependent receptor and an oxygenase in iron-dependent copper resistance in *Caulobacter crescentus*

Pauline Cherry[a], Hala Kasmo[a], Mauro Godelaine[a], Françoise Tilquin[a], Marc Dieu[b], Patsy Renard[b], Jean-Yves Matroule[a, 1]

[a] Research Unit in Microorganisms Biology (URBM), Department of Biology, Namur Research Institute for Life Sciences (NARILIS), University of Namur, Namur, Belgium

[b] MaSUN, Mass Spectrometry Facility, University of Namur, Namur, Belgium.

[1] corresponding author: jean-yves.matroule@unamur.be

References

[1] K. Sprouffske and A. Wagner, "Growthcurver: an R package for obtaining interpretable metrics from microbial growth curves," *BMC Bioinformatics*, vol. 17, no. 1, p. 172, Dec. 2016, doi: 10.1186/s12859-016-1016-7.

[2] J. Jumper *et al.*, "Highly accurate protein structure prediction with AlphaFold," *Nature*, vol. 596, no. 7873, pp. 583–589, Aug. 2021, doi: 10.1038/s41586-021-03819-2.

[3] E. F. Pettersen *et al.*, "UCSF ChimeraX : Structure visualization for researchers, educators, and developers," *Protein Science*, vol. 30, no. 1, pp. 70–82, Jan. 2021, doi: 10.1002/pro.3943.

[4] W. C. Nierman *et al.*, "Complete genome sequence of *Caulobacter crescentus*," *Proc. Natl. Acad. Sci. U.S.A.*, vol. 98, no. 7, pp. 4136–4141, Mar. 2001, doi: 10.1073/pnas.061029298.

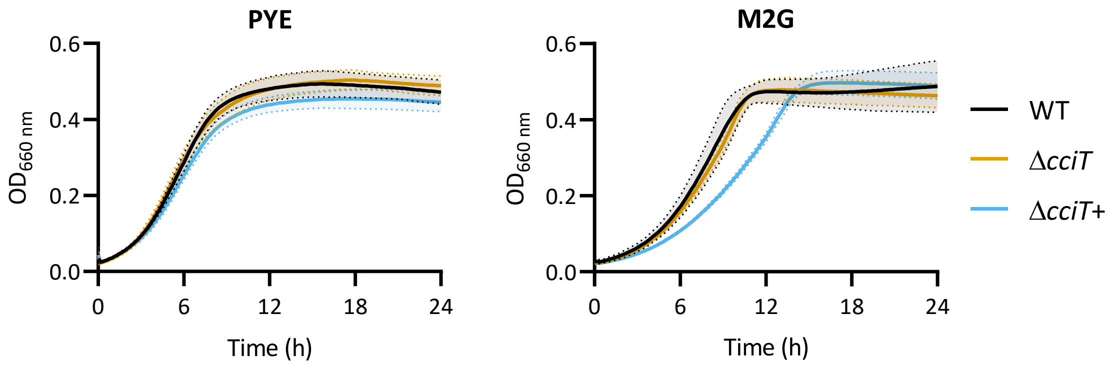

Figure S1. The deletion of *cciT* does not impact the fitness in control conditions. Growth profiles at an absorbance of 660 nm of WT, Δ*cciT*, and Δ*cciT*+ strains in PYE (top) and in M2G (bottom) media. Mean ± SD, at least three biological replicates.

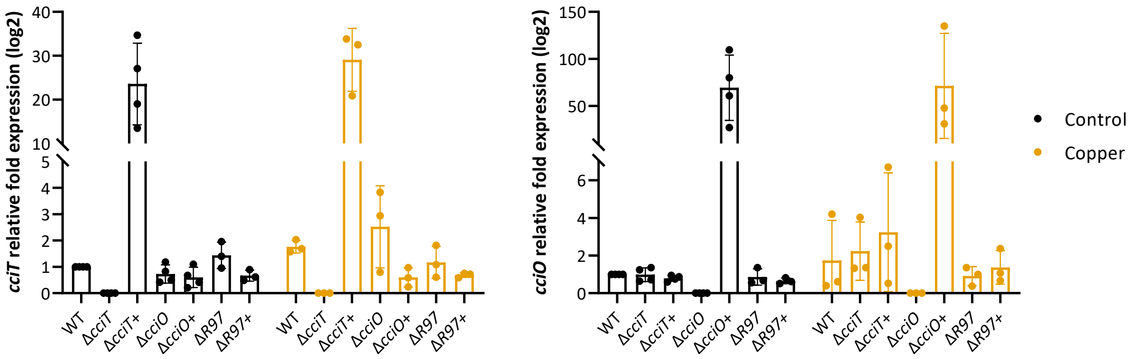

Figure S2. Expression levels of *tbcT* and *oxcT* genes in the studied genetic backgrounds. The ectopic expression of either *tbcT* or *oxcT* genes from the low copy plasmid pMR10 induces overexpression of said gene compared to the WT background. The strains were grown in M2G medium and subjected to Cu excess for 10 minutes. The deletion of *oxcT* does not impact the expression of the *tbcT* gene, or vice versa. Cu excess does not impact the expression levels as observed in control conditions. *mreB* gene has been used as a housekeeping gene. Biological replicates ≥ 3, technical replicates = 3, mean ± SD.

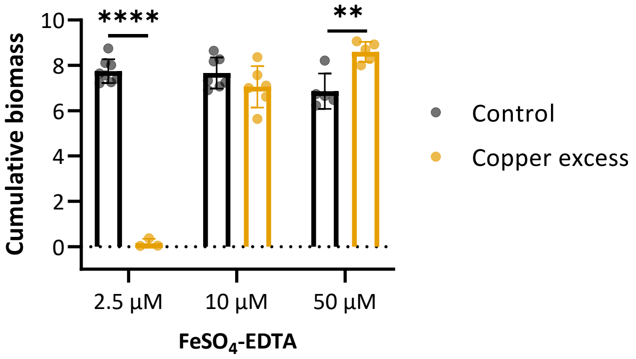

Figure S3. Effect of Fe concentration in the medium on the growth of *C. crescentus* with and without Cu excess. The area under the curve of the growth curves depicted on Fig. 3A was calculated using R package growthcurver [1]. Mean ± SD, at least three biological replicates. *p* values were calculated using ANOVA combined with Tukey multiple comparison test (***p* < 0.01, *****p* < 0.001).

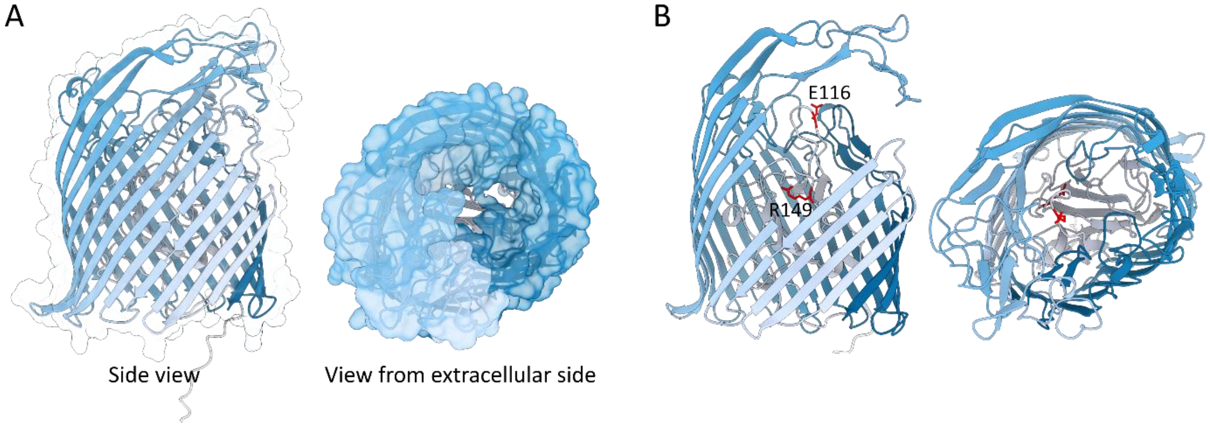

Figure S4. Structure prediction of CciT using AlphaFold [2]**. A.** TbcT is composed of 22 antiparallel β-strands forming a β-barrel, in which lies the globular plug domain. In white is the potential TonB box. The surface of the β-barrel has been represented, allowing the visualization of the entry of the substrate from the extracellular side. **B.** The residues potentially involved in substrate recognition, E116 and R149, are colored in red and the side chains are shown. Models were visualized using ChimeraX [3].

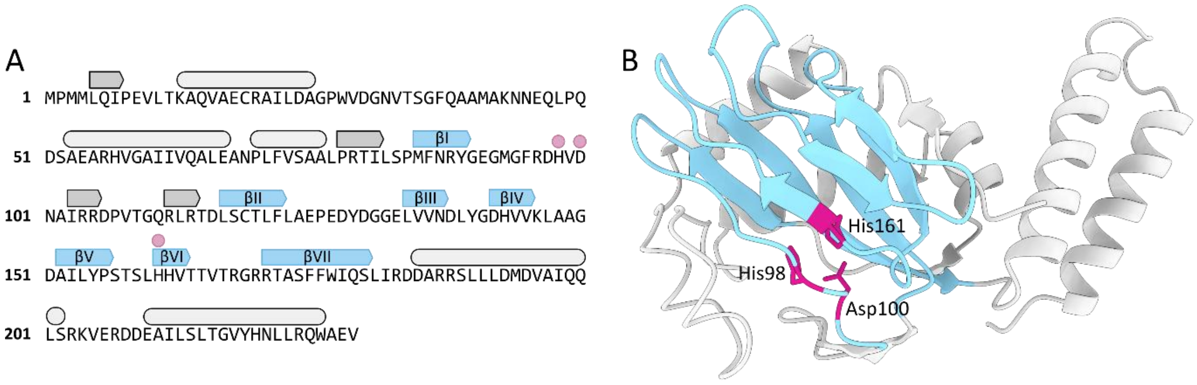

Figure S5. Structure prediction of CciO by AlphaFold [2]**. A and B.** The DSBH core fold is colored in light blue and the residues coordinating the Fe^{2+} cofactor are colored in purple. **A.** Schematic representation of the secondary structure of CciO, based on the 3D structure prediction. β-strands are represented by arrows and α-helices by rounded squares. The additional secondary structures are represented in light grey for α-helices and dark grey for β-strands. **B.** The DSBH fold adopts a squashed barrel conformation, supported by N-terminal secondary structures. The C-terminal α-helices are apart from the core fold. The model was visualized using ChimeraX [3].

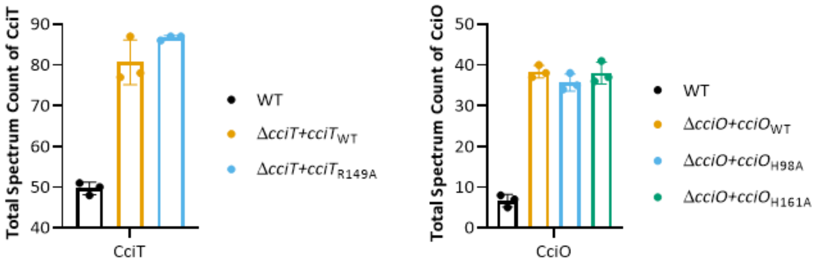

Figure S6. The ectopic expression of *cciT* and *cciO* leads to increased protein abundance. Normalized spectrum count of peptides in WT, Δ*cciT*+*cciT*$_{WT}$, Δ*cciT*+*cciT*$_{R149A}$, Δ*cciO*+*cciO*$_{WT}$, Δ*cciO*+*cciO*$_{H98A}$ and Δ*cciO*+*cciO*$_{H161A}$ strains grown in M2G medium, measured by LC-MS. Individual values and means represented.

Table S1. Bacterial strains and plasmids used in this study

Strains	Genotype and/or phenotype	Reference or source
Caulobacter crescentus		
WT	Wild-type NA1000, synchronizable variant of CB15	[4]
Δ*cciO*	Knock-out strain for *cciO* gene (CCNA_00027)	This study
Δ*cciO*+*cciO*$_{WT}$	Knock-out strain for *cciO* gene carrying a copy of *cciO* on the pMR10 under the control of the lac promoter; Kan^R	This study
Δ*cciO*+*cciO*$_{H98A}$	Knock-out strain for *cciO* gene carrying a copy of *cciO* with the point mutation H98A on the pMR10 under the control of the lac promotor; Kan^R	This study
Δ*cciO*+*cciO*$_{H161A}$	Knock-out strain for *cciO* gene carrying a copy of *cciO* with the point mutation H161A on the pMR10 under the control of the lac promotor; Kan^R	This study
ΔR97	Knock-out strain for the ncRNA (CCNA_R0097)	This study
ΔR97+R97	Knock-out strain for CCNA_R0097 ORF carrying a copy of R97 on the pMR10 under the control of the lac promoter; Kan^R	This study
Δ*cciT*	Knock-out strain for *cciT* gene (CCNA_00028)	This study
Δ*cciT*+*cciT*$_{wt}$	Knock-out strain for *cciT* gene carrying a copy of *cciT* on the pMR20 under the control of the lac promotor; Tet^R	This study
Δ*cciT*+*cciT*$_{R149A}$	Knock-out strain for *cciT* gene carrying a copy of *cciT* with the point mutation R149A on the pMR20 under the control of the lac promotor; Tet^R	This study
Δ*operon*	Knock-out strain for *cciO* and *cciT* genes, and ncRNA R97 (CCNA_00027-R97-28)	This study
Δ*operon*+*operon*	Knock-out strain for *cciO* and *cciT* genes, and ncRNA R97, carrying a copy of the whole operon under the control of the lac promotor; Kan^R	This study
ΔCCNA_00138	Knock-out strain for CCNA_00138 gene	This study
Δ*hutA*	Knock-out strain for *hutA* gene (CCNA_02277)	This study
ΔCCNA_03023	Knock-out strain for CCNA_03023 gene	This study
Plasmids		
pNPTs138	mobRP4$^+$ ori-R6K *sacB*; integrative vector in *C. crescentus* for in-frame deletions; Kan^R	
pMR10	Low copy and replicative vector in *C. crescentus*; Kan^R	
pMR20	Derivative of pMR10; Tet^R	
